# Update on the recent development of allosteric modulators for adenosine receptors and their therapeutic applications

**DOI:** 10.3389/fphar.2022.1030895

**Published:** 2022-10-05

**Authors:** Silvia Pasquini, Chiara Contri, Martina Cappello, Pier Andrea Borea, Katia Varani, Fabrizio Vincenzi

**Affiliations:** ^1^ Department of Chemical, Pharmaceutical and Agricultural Sciences, University of Ferrara, Ferrara, Italy; ^2^ Department of Translational Medicine, University of Ferrara, Ferrara, Italy; ^3^ University of Ferrara, Ferrara, Italy

**Keywords:** adenosine, allosteric modulators, GPCRs, adenosine receptors, drug development

## Abstract

Adenosine receptors (ARs) have been identified as promising therapeutic targets for countless pathological conditions, spanning from inflammatory diseases to central nervous system disorders, from cancer to metabolic diseases, from cardiovascular pathologies to respiratory diseases, and beyond. This extraordinary therapeutic potential is mainly due to the plurality of pathophysiological actions of adenosine and the ubiquitous expression of its receptors. This is, however, a double-edged sword that makes the clinical development of effective ligands with tolerable side effects difficult. Evidence of this is the low number of AR agonists or antagonists that have reached the market. An alternative approach is to target allosteric sites *via* allosteric modulators, compounds endowed with several advantages over orthosteric ligands. In addition to the typical advantages of allosteric modulators, those acting on ARs could benefit from the fact that adenosine levels are elevated in pathological tissues, thus potentially having negligible effects on normal tissues where adenosine levels are maintained low. Several A_1_ and various A_3_AR allosteric modulators have been identified so far, and some of them have been validated in different preclinical settings, achieving promising results. Less fruitful, instead, has been the discovery of A_2A_ and A_2B_AR allosteric modulators, although the results obtained up to now are encouraging. Collectively, data in the literature suggests that allosteric modulators of ARs could represent valuable pharmacological tools, potentially able to overcome the limitations of orthosteric ligands.

## Introduction

Adenosine is a fundamental component of human physiology. It is a major constituent of nucleic acids, of life’s “energy currency” and signaling molecule adenosine triphosphate (ATP), as well as a ubiquitous cell function modulator itself. Adenosine acts as an autocrine/paracrine mediator with a short half-life whose low extracellular levels in healthy tissues are maintained mostly by rapid cellular uptake and cytosolic metabolism by adenosine deaminase or adenosine kinase (Haskó et al., 2008). However, following tissue injury, cells release large amounts of ATP, which is then converted to adenosine by ecto-nucleotidases. Generally, the resulting increased concentration of adenosine has largely beneficial effects in acute pathological conditions by restoring tissue homeostasis (Borea et al., 2016), while its chronic overproduction can be detrimental and cause inflammation, fibrosis, and organ damage (Borea et al., 2017). Adenosine triggers its effects through the interaction with four G-protein coupled receptors (GPCRs), named A_1_, A_2A_, A_2B_, and A_3_ adenosine receptors (ARs). Some of the biological functions of adenosine include, but are not limited to, regulation of neurotransmitter release, neuronal excitability, heart rate and contractility, blood flow, platelet aggregation, inflammation and immune system responses, wound healing, and metabolic processes (Borea et al., 2018). In addition to the several physiological effects of adenosine, its receptor-mediated signaling has many documented effects on the progression of countless pathological states (Karmouty-Quintana et al., 2013). Among the main ones, modulation of adenosine receptors has been indicated as a promising therapeutic strategy in pathological states such as cancer (Vijayan et al., 2017; Allard et al., 2020), cardiovascular diseases (Reiss et al., 2019), pain (Vincenzi et al., 2020a), neurological/neurodegenerative diseases (Blum et al., 2018; Sebastião et al., 2018; Jenner et al., 2021; Merighi et al., 2021), neuropsychiatric disorders (Pasquini et al., 2022), inflammatory diseases (Pasquini et al., 2021; Antonioli et al., 2022), respiratory diseases (Caruso et al., 2013), ocular diseases (Spinozzi et al., 2021), diabetes, and other metabolic disorders (Antonioli et al., 2015; Sanni and Terre’Blanche, 2021). Despite this encouraging profusion of experimental evidence, relatively few adenosinergic system-based drugs have so far achieved clinical approval. When looking for accountability for this lack of finalization, this cannot be attributed to the lack of highly affine and selective ligands, as the search for new ligands has been quite productive (Jacobson et al., 2021; IJzerman et al., 2022), but rather the redundancy of adenosine signaling, the agonist-dependent receptor desensitization, and the broad expression of ARs provide the biggest challenges (Peleli et al., 2017). As a result of these drawbacks, most attempts to test orthosteric AR ligands in clinical trials have failed due to inefficiency or serious and unfavorable side effects. Different strategies were explored to overcome the above-mentioned obstacles, including partial agonists (Greene et al., 2016; Voors et al., 2019), indirect receptor targeting (Kutryb-Zajac et al., 2020; Wang et al., 2021), prodrugs (Suresh et al., 2020), multi-target drugs (Huang et al., 2011), but one of the most promising seems to be allosteric modulation. By affecting endogenous agonist affinity and/or efficacy, a positive allosteric modulator (PAM) is an allosteric ligand that enhances an agonist-mediated receptor response, while a negative allosteric modulator (NAM) attenuates activity (Gentry et al., 2015). Other classes include neutral allosteric ligands (NAL) that bind at the allosteric site without affecting receptor or orthosteric ligand activity and allosteric agonists, ligands that directly activate the receptors from the allosteric site even in the absence of an orthosteric agonist. Traditionally, GPCRs have been targeted using compounds that bind to the orthosteric site. Allosteric ligands, binding at sites that are topologically distinct from the orthosteric sites, have expanded the ways to manipulate GPCR functionality, providing several pharmacological advantages and potential therapeutic benefits (Wootten et al., 2013). Due to the reduced evolutionary pressure that would ordinarily be necessary to maintain an orthosteric binding pocket capable of accepting the endogenous ligand, allosteric sites are less conserved among related receptor subtypes (Wild et al., 2014). Furthermore, since allosteric modulators may cause a variety of conformational changes in GPCR structures, they can be rationally tailored to create a strong biased signaling response from a GPCR triggered by an otherwise non-biased orthosteric ligand (Wold and Zhou, 2018). By imparting biased modulation upon orthosteric agonists, these allosteric modulators have the ability to only enhance therapeutically relevant signaling while preventing on-target side effects (Gao et al., 2011; Slosky et al., 2021). Apart from allosteric agonists, allosteric modulators such as PAMs and NAMs only have an effect in the presence of orthosteric ligands and can enhance or decrease receptor activation induced by endogenous agonists. Therefore, they act more physiologically and are predicted to have fewer adverse effects and tolerance-inducing consequences than orthosteric ligands. A particularly crucial element in the case of the short-lived molecule adenosine is the ability of PAMs and NAMs to finely tune its activity by following the spatiotemporal distribution of its extracellular concentration. Another important advantage is the reciprocal communication with the orthosteric domain: the allosteric modulator exerts an effect on the binding of the endogenous ligand, but the latter can also affect the binding of the modulator. This mechanism supports the selectivity of allosteric ligands, especially under conditions where there is a pathology-dependent alteration in the concentration of the endogenous agonist at a particular site (Draper-Joyce et al., 2021). This review summarizes the advances in the development of ARs allosteric modulators (Table 1) that may provide support for their use as new therapeutic options.

**TABLE 1:** Selected *in vitro* and *in vivo* studies on AR allosteric modulators.

Compound	Type	*In vivo* models	Main results	References
PD 81,723 ((2-amino-4,5-dimethylthiophen-3-yl)-[3-(trifluoromethyl)phenyl]methanone) 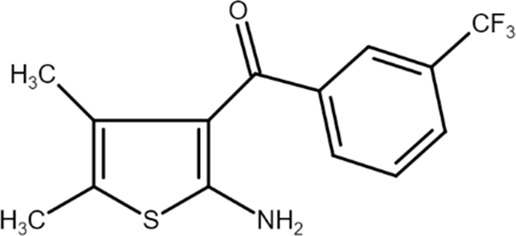	A_1_AR PAM	Hyperglycemic cerebral ischemia and reperfusion in rats	Hippocampal injury reduction and Morris water maze performance improvement	Meno et al. (2003)
		Renal ischemia-reperfusion injury in mice	Renal tubular necrosis and inflammation reduction	Park et al. (2012)
T62 ((2-amino-4,5,6,7-tetrahydro-1-benzothiophen-3-yl)-(4-chlorophenyl)methanone) 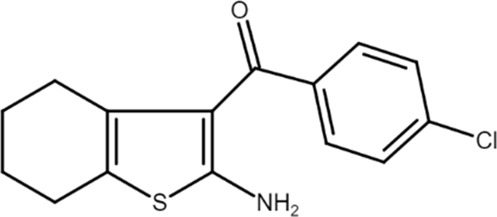	A_1_AR PAM	Spinal nerve ligation-induced mechanical hypersensitivity in rats	Mechanical hypersensitivity decrease	Pan et al. (2001)
		Carrageenin-induced thermal hypersensitivity in rats	Thermal hypersensitivity decrease	Li et al. (2003)
		Plantar incision-induced hypersensitivity in rats	Mechanical hypersensitivity reduction	Obata et al. (2004)
TRR469 ((2-Amino-4-[(4-(phenyl)piperazin-1-yl)methyl]-5-(4-fluorophenyl)thiophen-3-yl)-(4-chlorophenyl)methanone) 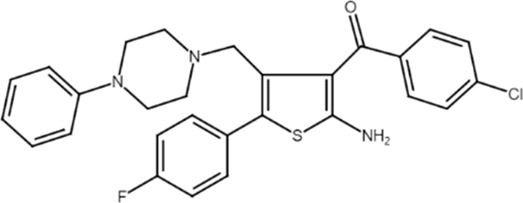	A_1_AR PAM	Formalin and writhing tests, and streptozotocin-induced diabetic neuropathic pain in mice	Acute and chronic pain reduction	Vincenzi et al. (2014)
		Anxiety behavioral paradigms in mice	Anxiolytic-like effects	Vincenzi et al. (2016)
		Glutamate-induced injury in PC12 cells	Cell death, caspase activation, ROS production, and mitochondrial membrane potential loss abrogation	Vincenzi et al. (2020b)
VCP333 (*tert*-butyl 2-amino-3-(4-chlorobenzoyl)-7,8-dihydro-4H-thieno [2,3-d]azepine-6(5H)-carboxylate) 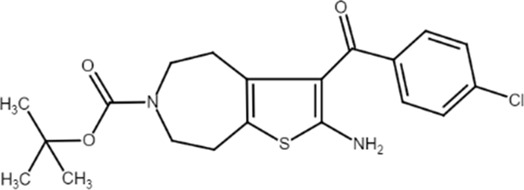	A_1_AR PAM	Ischemia-reperfusion in murine isolated hearts	Cardiac function improvement and myocardial cell death reduction	Butcher et al. (2013)
VCP171 ((2-amino-4-[3-(trifluoromethyl)phenyl)thiophen-3-yl]-phenylmethanone) 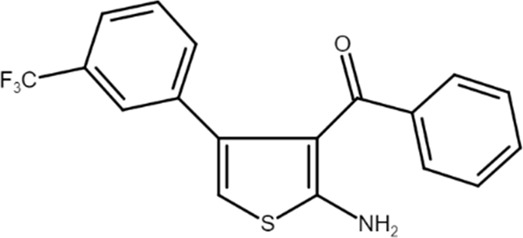	A_1_AR PAM	Partial nerve-injury neuropathic pain in rats	eEPSC amplitude of nerve-injury inhibition	Imlach et al. (2015)
MIPS521 (2-amino-4-(3,5-bis(trifluoromethyl)phenyl)thiophen-3-yl) (4-chlorophenyl)methanone) 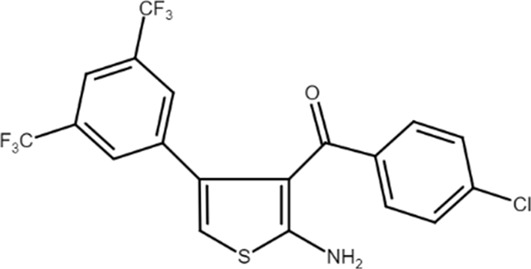	A_1_AR PAM	Partial nerve ligation-induced neuropathic pain in rats	Spinal nociceptive signaling and mechanical allodynia reduction	Draper-Joyce et al. (2021)
AEA061 (Chemical structure not disclosed)	A_2A_AR PAM	LPS-induced endotoxemia in mice	Plasma TNF-α and MCP-1 level reduction, and IL-10 increase	Welihinda and Amento (2014)
		LPS-stimulated splenic monocytes/macrophages	Cytokine/chemokine reduction	Welihinda and Amento (2014; Welihinda et al. (2018)
		Imiquimod- or IL-23-induced psoriasis-like dermatitis in mice	Clinical score and cytokine expression reduction	Welihinda et al. (2022)
A2AR PAM-1 (3,4-difluoro-2-((2-fluoro-4-iodophenyl)amino)benzoic acid) 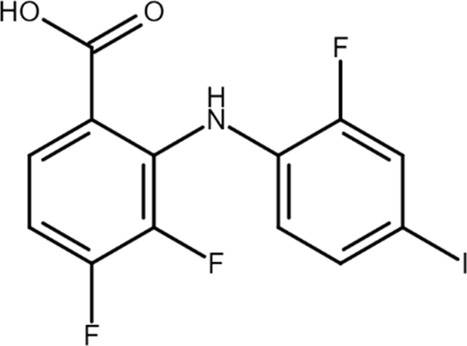	A_2A_AR PAM	EEG/EMG electrodes implanted in mice	Slow-wave sleep induction	Korkutata et al. (2017, 2019)
KI-7 (2-(1-benzyl-1H-indol-3-yl)-2-oxo-N-phenylacetamide) 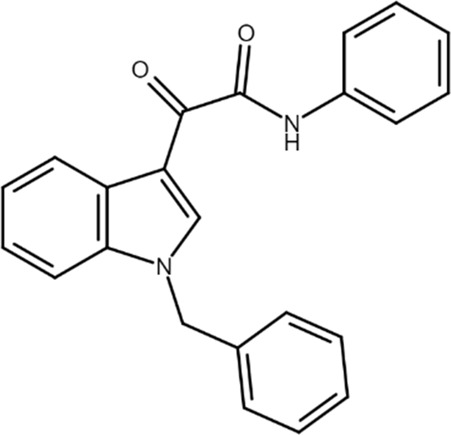	A_2B_AR PAM	Mesenchymal stem cells	Osteoblast differentiation and survival increase	Trincavelli et al. (2014a)
Compound 9 (N-(4-chlorobenzyl)-2-(benzofuran-2-yl)glyoxylamide) 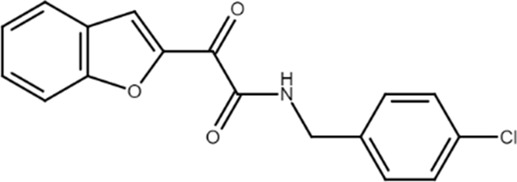	A_2B_AR PAM	Mesenchymal stem cells	Matrix mineralization stimulation	Barresi et al. (2021a)
LUF6000 (CF602) 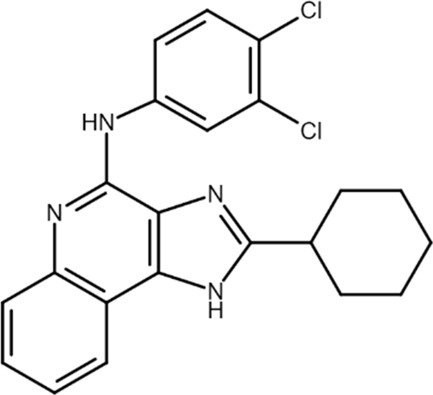	A_3_AR PAM	Adjuvant-induced arthritis in rats	Arthritis clinical score reduction	Cohen et al. (2014)
		Monoiodoacetate-induced osteoarthritis in rats	Knee swelling and edema decrease	
		Concanavalin A-induced liver inflammation in mice	Serum glutamic pyruvate transaminase and serum glutamic oxaloacetic transaminase decrease	
		Diabetic erectile dysfunction in rats	Intracavernosal pressure increase	Itzhak et al. (2022)
LUF6096 (N-[2-(3,4-dichloroanilino) quinolin-4-yl]cyclohexane carboxamide) 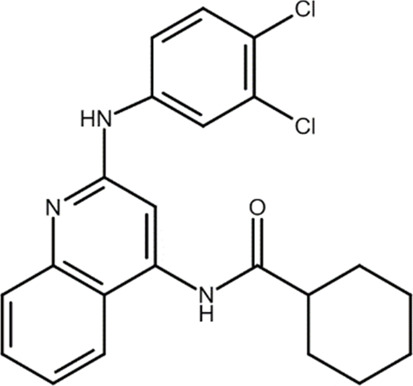	A_3_AR PAM	Myocardial ischemia/reperfusion injury in dogs	Infarct size reduction	Du et al. (2012)

## Allosteric modulation of ARs

### A_1_AR allosteric modulators

A_1_ARs are widespread and implicated in many physiological mechanisms, therefore they are regarded as a prominent drug target for different diseases. Adenosine through A_1_ARs exerts sedative, anticonvulsant, anxiolytic, and locomotor depressant effects (Varani et al., 2017). Furthermore, the heart rate and rhythm, the conduction speed in the atrioventricular node, and cardiac muscle contraction are negatively controlled by A_1_ARs (Deb et al., 2019; Jacobson et al., 2019). In particular, A_1_AR agonists mediate cardioprotection through the inhibition of norepinephrine release (Dinh et al., 2017). An important role of A_1_ARs is in nociception, due to their location in peripheral sensory nerve terminals in the spinal cord dorsal horn and in supraspinal pain-processing structures (Sawynok, 2016; Vincenzi et al., 2020a). Many studies have been conducted to exploit the therapeutic potential of these receptors, but the development of orthosteric agonists has been hampered by several drawbacks, the main ones being cardiac side effects and receptor desensitization. An alternative strategy to exploit the positive effects of A_1_AR stimulation is allosteric modulation. Much research effort in recent decades has been devoted to the synthesis and *in vitro* and *in vivo* evaluation of A_1_AR PAMs (Romagnoli et al., 2015b; Jacobson and Gao, 2016). The first and most extensively studied class of compounds synthesized are the benzoylthiophene derivatives, the prototype of which is PD 81,723 (Bruns et al., 1990). Different studies revealed a potential application of PD 81,723 in ischemic injury (Meno et al., 2003; Park et al., 2012). Another extensively studied compound belonging to this class of modulators is T62 (Baraldi et al., 2000). It was effective in reducing nociception and hypersensitivity in animal models of neuropathic pain (Pan et al., 2001; Li et al., 2002, 2003; Obata et al., 2004). It was also used in a phase II clinical trial for postherpetic neuropathic pain. However, the study was abandoned due to a lack of efficacy and the presence of transient high levels of liver transaminase in some patients (Giorgi and Nieri, 2013). Subsequently, numerous other derivatives were discovered, endowed with greater allosteric activity (Romagnoli et al., 2008, 2012, 2013, 2014; 2015a). Of these, TRR469 was selected for *in vivo* studies. TRR469 has been reported to have an analgesic effect comparable to that of morphine in animal models of both acute and neuropathic pain without showing the side effects typical of orthosteric A_1_AR agonists such as locomotor disturbances or sedation (Vincenzi et al., 2014). This compound also proved effective as an anxiolytic in several mouse models of anxiety with an effect comparable to that of diazepam but without the locomotor side effects typical of benzodiazepines (Vincenzi et al., 2016). Also noteworthy is the protective effect of TRR469 found in an *in vitro* model of glutamate-induced cytotoxicity in neuronal cells (Vincenzi et al., 2020b). Another series of 2-amino-3-benzoylthiophene A_1_AR PAMs were synthesized (Aurelio et al., 2009), including VCP171, whose *in vivo* analgesic effect in a model of neuropathic pain proved weaker than that of the orthosteric A_1_AR agonist, but which nevertheless has greater therapeutic potential due to fewer side effects, particularly in tissues with higher adenosine concentrations or A_1_AR tone (Imlach et al., 2015). Instead, VCP333 has been shown to improve cardiac function and reduce cardiomyocyte death following cardiac ischemia (Butcher et al., 2013). The most recently synthesized is MIPS521, an A_1_AR PAM that has shown analgesic effects in models of neuropathic pain by being able to modulate the high concentrations of adenosine present. Interestingly, a new binding pocket was identified by studying the structure of the A_1_AR bound to adenosine, MIPS521, and the Gi protein. This lead to hypothesize that the modulator also exerts its effects by stabilizing the adenosine-receptor-G protein complex (Draper-Joyce et al., 2021). Recently, a multisite binding model for A_1_AR allosteric modulation has been proposed. It predicts that there are several extracellular sites capable of binding the modulator, not just a distinct pocket generally located on the second extracellular loop (Deganutti et al., 2021).

### A_2A_AR allosteric modulators

Adenosine, mainly through the activation of A_2A_ARs expressed in peripheral immune cells, represents a potent inflammatory self-limiting factor (Antonioli et al., 2022). Depending on the pathology, this can have both positive and negative impacts. On the positive side, A_2A_AR activation is potentially useful for the treatment of autoimmune and inflammatory diseases (Vincenzi et al., 2013), as evidenced by the fact that the anti-inflammatory and immunosuppressive effects of methotrexate, a gold standard for the treatment of rheumatoid arthritis, as well as some of the anti-inflammatory effects of sulfasalazine, are mediated by adenosine (Cronstein and Sitkovsky, 2017). Although A_2A_AR agonists may be effective in the treatment of inflammatory illnesses, they are likely to have too many adverse effects to be tolerated, mainly owing to their significant hypotensive effect. One alternative approach that could potentially circumvent the agonist-related side effects while enhancing the potent anti-inflammatory action of adenosine is represented by A_2A_AR PAMs. On the negative side of adenosine-mediated inflammation suppression, many solid tumors escape immune response by increasing the concentration of adenosine in the tumor microenvironment. Both animal studies and clinical trials have shown that blocking A_2A_AR can induce tumor regression (Sun et al., 2022). Although not yet tested, one can speculate that A_2A_AR NAM could potentially counteract in a spatial-selective manner the tumor-increased adenosine immunosuppressive action. In the CNS, blockade of A_2A_ARs is indicated, with varying degrees of preclinical and clinical evidence, as a promising therapeutic strategy for Parkinson’s disease, supported by the recent approval of the antagonist istradefylline as add-on therapy (Chen and Cunha, 2020), but also for Alzheimer’s disease (Merighi et al., 2022), acute brain dysfunction (Cunha, 2016), and some neuropsychiatric disorders such as fragile X syndrome, depression, and anxiety (Domenici et al., 2019).

Unfortunately, only a small number of A_2A_AR allosteric modulators have been reported so far. Some N^6^-1,3-diphenylurea derivatives of 2-phenyl-9-benzyladenines and 8-azaadenines have been suggested to act as allosteric modulators at the A_2A_ARs (Giorgi et al., 2008). Later, using a fragment screening technique, some PAMs and NAMs of ARs were identified. In particular, ZB1854 potentiated the action of the A_2A_AR agonist CGS 21680, thereby behaving as a PAM (Chen et al., 2012). A compound denoted as AEA061 increased adenosine’s anti-inflammatory properties by allosterically enhancing its activity at A_2A_ARs in the lipopolysaccharide (LPS)-induced mouse model of inflammation (Welihinda and Amento, 2014). In a subsequent work, AEA061 was also shown to enhance inosine-mediated A_2A_AR activation and consequent inhibition of pro-inflammatory cytokine and chemokine production in splenic monocytes (Welihinda et al., 2018). Very recently, AEA061 also reduced clinical scores and cytokine expression in two different models of psoriasis-like dermatitis induced by imoquimod or IL-23 (Welihinda et al., 2022). Another A_2A_AR PAM, named A_2A_R PAM-1, increased the total amount of slow wave sleep, from which individuals with insomnia might benefit, without affecting blood pressure, heart rhythm, and body temperature as the agonist CGS21680 did (Korkutata et al., 2017, 2019).

### A_2B_AR allosteric modulators

The A_2B_AR is widely expressed in organs such as the bladder, intestine, and lung, as well as in various cell types such as fibroblasts, smooth muscle, endothelial, immune, and alveolar epithelial cells (Borea et al., 2018). Of all the ARs, the A_2B_ subtype is the least characterized from a pharmacological point of view. It has been proposed as a potential target in acute lung injury, as its activation with the agonist BAY 60–6,583 led to a reduction in inflammation and pulmonary edema, and an increase in alveolar fluid clearance (Eckle et al., 2013; Hoegl et al., 2015; Wang et al., 2020). Recently, Gnad and others found that activation of A_2B_ARs restores muscle and brown fat function in elderly and obese mice to that of young, lean animals, establishing its anti-aging and anti–obesity potential (Gnad et al., 2022). In addition, it has been suggested that A_2B_ARs have therapeutic potential in bone diseases, as their activation appears to promote osteoblast differentiation and bone formation (Carroll et al., 2012; Corciulo et al., 2016).

The first class of allosteric modulators for the A_2B_AR, a series of 1-benzyl-3-ketoindoles, was serendipitously discovered (starting from a scaffold previously used to develop benzodiazepine receptor ligands) and consisted of three PAM and four NAM (Taliani et al., 2013; Trincavelli et al., 2014b). Subsequently, one of these A_2B_AR PAMs, denoted as KI-7, was shown to enhance the effects of adenosine and synthetic A_2B_AR agonists in the differentiation of mesenchymal stem cells (MSC) to osteoblasts while also increasing differentiated osteoblast viability (Trincavelli et al., 2014a). More recently, a series of novel derivatives chemically related to those previously synthesized has been reported. One of these compounds, a benzofurane derivative that was confirmed to behave as A_2B_AR PAM, stimulated matrix mineralization in MSC, making it a lead structure for the synthesis of new compounds with anti-osteoporosis properties (Barresi et al., 2021a; 2021b).

Since A_2B_AR blockade may represent a promising approach for the treatment of some diseases, such as in cancer immunotherapy (Gao and Jacobson, 2019), A_2B_AR NAM could also result in a valuable pharmacological resource. Interestingly, the well-known selective A_2B_AR antagonist PSB 603 was recently suggested to act as a NAM, at least in A_2B_-mediated cAMP accumulation in HEK 293 cells (Goulding et al., 2018).

### A_3_AR allosteric modulators

A_3_AR is expressed in the brain, heart, testis, lung, placenta, uterus, kidneys, spleen, liver, bladder, and proximal colon, but, while low expression is found in normal cells, this receptor subtype is overexpressed in immune and cancer cells (Gessi et al., 2008). The activation of A_3_AR mediates anti-inflammatory, antitumor, and anti-ischemic beneficial effects, showing a therapeutic potential for the treatment of inflammatory diseases, such as rheumatoid arthritis and psoriasis, hepatitis, cancer, glaucoma, cardiovascular diseases, and cerebral ischemia (Borea et al., 2015).

In addition to some selective A_3_AR agonists, several series of allosteric modulators, mainly PAMs, have also been developed, representing an alternative approach for the treatment of those aforementioned diseases in which A_3_AR activation appears to be a promising therapeutic strategy (Gao et al., 2001, 2002; Göblyös et al., 2006; Heitman et al., 2009; Kim et al., 2009). However, as opposed to orthosteric agonists, A_3_AR PAMs have the benefit of being able to target regions where adenosine levels are elevated, such as tumor and inflammatory sites, with low or no effects on normal tissues where adenosine levels are low.

Among the most well-known A_3_AR PAMS are the LUF6000 and LUF6096. LUF6000 is an imidazoquinolinamine PAM at the A_3_AR that showed anti-inflammatory effects in a rat adjuvant-induced arthritis model, inhibited monoiodoacetate-induced osteoarthritis development, and exhibited protective effects in a liver inflammation model of acute hepatitis in mice. At the molecular level, LUF6000 administration resulted in a marked deregulation of the NF-κB signaling pathway (Cohen et al., 2014). Itzhak and co-workers evaluated the effect of LUF6000 (also known as CF602) on resolving erectile dysfunction (ED) in a diabetic ED rat model. CF602 increased intracavernosal pressure, endothelial nitric oxide synthase (eNOS), and vascular endothelial growth factor (VEGF) levels, improving erectile function (Itzhak et al., 2022). This compound may thus provide an alternative treatment for phosphodiesterase 5 (PDE5) inhibitors, which are usually employed in ED therapy, considering that half of the patients with diabetes do not respond to PDE5 inhibitors.

In addition, LUF6096, which is structurally similar to LUF6000, reduced infarct size in a barbital-anesthetized dog model of myocardial ischemia/reperfusion injury. The infarct size reduction was equally evident when LUF6096 was administered in two doses before coronary artery occlusion and immediately before reperfusion or a single dose immediately before reperfusion (Du et al., 2012).

Studies conducted by Lane and others suggest that the endocannabinoid two- arachidonylglycerol (2-AG) acts as a NAM at the A_3_AR. This evidence may be especially important in certain pathological conditions like cerebral ischemia when levels of 2-AG are elevated and could interact with A_3_AR expressed in astrocytes and microglia (Lane et al., 2010).

## Conclusion

GPCR allosteric modulators are promising therapeutic agents. By altering the receptor conformation, they potentiate or attenuate the effect of the endogenous agonist, acting more physiologically than orthosteric ligands and offering spatiotemporal selectivity. The adenosinergic system, making use of a short-lived autocrine/paracrine mediator, represents an ideal situation to take advantage of the benefits of allosteric modulation. The available preclinical results are encouraging, and there is hope for an acceleration that may lead to the clinical use of allosteric modulators of ARs. Nevertheless, no allosteric modulator has entered clinical trials to date, underlining the challenges in the discovery and development of this class of compounds. Allosteric sites generally have a shallow structure-activity relationship and are often unknown or difficult to discover as they are only accessible in specific protein conformations. The fact that allosteric sites are less evolutionarily conserved than orthosteric ones can lead to species differences that can hamper their validation. Furthermore, allosteric modulators have a high propensity for molecular switching and can show complex *in vivo* pharmacology. Despite these challenges to identifying, validating, and developing allosteric modulators for GPCRs, they have the potential to become one of the most highly effective and minimally toxic pharmacological agents.
